# Molecular Targets of Active Anticancer Compounds Derived from Marine Sources

**DOI:** 10.3390/md16050175

**Published:** 2018-05-22

**Authors:** Xiaoping Song, Ying Xiong, Xin Qi, Wei Tang, Jiajia Dai, Qianqun Gu, Jing Li

**Affiliations:** 1 Key Laboratory of Marine Drugs of Minister of Education of China, School of Medicine and Pharmacy, Ocean University of China, Qingdao 266071, China; xiaopingsong@126.com (X.S.); xiongyyyyy@163.com (Y.X.); qxin@163.com (X.Q.); iamtangwei1992@sina.com(W.T.); 15898863889@163.com (J.D.); guqianq@ouc.edu.cn (Q.G.); 2 Qingdao National Laboratory for Marine Science and Technology, Qingdao 266237, China

**Keywords:** marine sources, anticancer compounds, molecular targets

## Abstract

Over the past decades, a number of novel compounds, which are produced in the marine environment, have been found to exhibit the anticancer effects. This review focuses on molecular targets of marine-derived anticancer candidates in clinical and preclinical studies. They are kinases, transcription factors, histone deacetylase, the ubiquitin-proteasome system, and so on. Specific emphasis of this review paper is to provide information on the optimization of new target compounds for future research and development of anticancer drugs, based on the identification of structures of these target molecules and parallel compounds.

## 1. Introduction

According to the latest published cancer statistics by the American Cancer Society [1,2], despite the fact that the overall cancer incidence rate declines year by year due to rapid development of novel anticancer agents, cancer remains an impending public health problem and leads to a huge burden around the world. 

Due to the harsh and competitive conditions in the marine environment, the compounds produced by marine organism exhibit unique structural scaffolds [3]. Especially, it is noticeable that marine active natural products can form complex and elaborate three-dimensional structures during the process of biosynthesis and bind with the receptor molecules of the drug in the form of reticular non-covalent interaction [4]. Meanwhile, highly active functional groups in the molecular structure, such as epoxy group, lactone ring, lactam, sulfate, etc., can bind to different molecular targets in the form of a covalent linkage and exert various biological functions [5]. 

**Figure 1:**
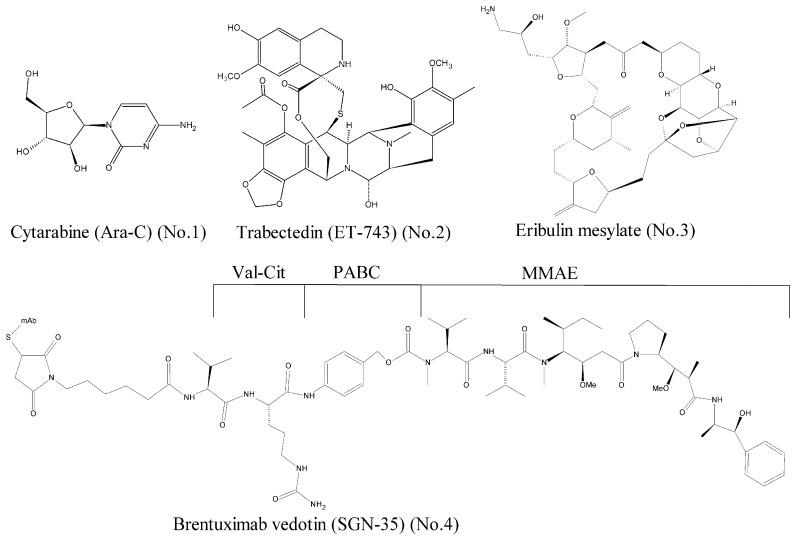
Marine-derived anticancer drugs approved by the FDA.

Over the past decades, a large number of marine-derived compounds have been screened, and a wide range of activities, such as antiviral, antibacterial, antitumor, antidiabetic, and anti-inflammatory, have been reported [6]. According to the data of National Institutes of Health, the anti-tumor activity rate of marine compounds is far greater than that of terrestrial compounds [7]. To our best knowledge, seven marine-derived drugs have been approved by the Food and Drug Administration (FDA) so far, and four of them are antitumor agents [8]. As shown in Figure 1, they are the cytarabine (Ara-C) [9] (No. 1), trabectedin (ET-743) [10] (No. 2), eribulin mesylate [11] (No. 3), and brentuximab vedotin (SGN-35) [12] (No. 4).

Previous reports on the anti-tumor mechanisms of marine-derived compounds mostly focused on the incorporation of these compounds into DNA and prevention of DNA synthesis (such as trabectedin), inhibition of DNA topoisomerase (such as cytarabine), affection of microtubule polymerization (such as eribulin mesylate), etc. Such compounds have cytotoxic effects and interfere with all rapidly divided cells with poor selectivity and a high-risk of toxic side effects. In recent years, as the interest of cancer research has shifted from the traditional cytotoxic drugs to molecule targeted antitumor drugs, an increasing number of leading compounds targeting abnormal molecules within tumor cells has been identified. These abnormal molecules are overexpressed or are mutant in the progression of cancer, including kinases, transcription factors, histone deacetylase, the ubiquitin-proteasome system, and so on.

This review focuses on molecular targets that have been reported to directly interact with marine-derived anticancer candidates in preclinical and clinical studies. It is expected to provide useful information on the identification of newly targeted compounds and their molecule optimization in the future, based on the identification of the structures of these compounds.

## 2. Molecular Targets of Marine-Derived Anticancer Candidates

### 2.1. Targeting the Kinases Related to Cell Survival and Proliferation Signaling Pathway

It is always a promising strategy to target relevant oncogene kinases of signaling pathways that are related to tumorigenesis and tumor progression. Increasing number of marine-derived compounds, which target the kinases, have been enrolled as anticancer candidates in vitro or in vivo models [13]. 

#### 2.1.1. Protein Kinase C (PKC)

**Figure 2:**
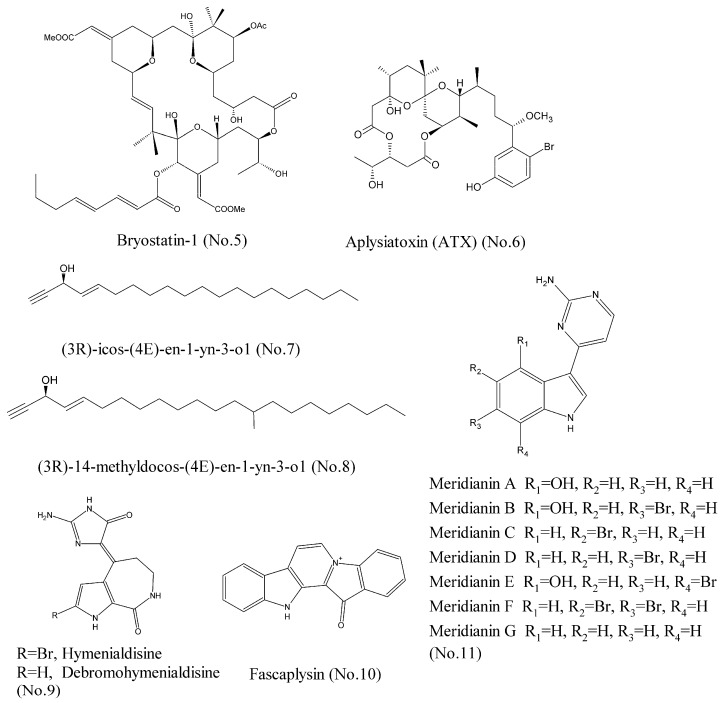
Compounds targeting PKC, IGF-1R, and CDKs.

For a long time, many studies have considered that protein kinase C (PKC) is an oncogene that promotes cancer progression. Therefore, many PKC inhibitors have been developed to counteract PKC kinase [14]. However, the latest research completely overthrows our previous understanding, and points out that PKC family, including cPKC (α, β, γ), nPKC (δ, ε, η), and aPKC (ζ) isozymes, function as tumor suppressors. This indicates that it is beneficial to search for the compounds that can activate PKC isozymes [15]. Bryostatin-1 (No. 5) (Figure 2), a highly-oxygenated macrolide with a unique polyacetate backbone, was originally isolated from marine bryozoan *Bugula neritina*. It was reported that bryostatin-1 activated PKC isozymes, specifically PKCα and PKCϵ at sub-nanomolar concentrations [16]. Wender et al. [17] proposed that bryostatin macrolactones exhibited high affinities for PKC isozymes, because they could compete with phorbol ester for the binding site on PKC and stimulate kinase activity in vitro and in vivo. Furthermore, in the past ten years, more than 20 clinical trials have been conducted with bryostatin-1 in monotherapy or in combination with cytotoxic drugs against various cancer types such as sarcoma, melanoma, ovaria, cervical, neck and head carcinoma, esophageal, gastric, pancreatic, and renal cell carcinoma, as well as leukemia, etc. [18]. Aplysiatoxin (ATX) [19] (No.6) (Figure 2), which was isolated from sea hare and cyanobacteria, was found to bind to activate protein kinase C (PKC) isozymes and lead to anti-proliferative activity against human cancer cell lines, suggesting that it could be used as a leading compound for development of anticancer drugs.

#### 2.1.2. Insulin-Like Growth Factor-1 Receptor (IGF-1R)

The insulin-like growth factor-1 receptor (IGF-1R) has become a potential therapeutic target for cancer [20]. IGF-1R signaling is transduced through two main pathways: (1) the RAS/RAF/MAP kinase pathway and (2) the phosphoinositide-3 kinase (PI3K)/Akt pathway. They are involved in tumor cell proliferation, survival, and invasion [21]. Several inhibitors of IGF-1R, including monoclonal antibodies and small molecule tyrosine kinase inhibitors, have entered clinical development for the treatment of solid tumors, including non-small cell lung cancer (NSCLC), small cell lung cancer (SCLC), and ovarian carcinoma (OC) [22]. Zovko et al. [23] recently characterized two acetylene alcohols: (3R)-icos-(4E)-en-1-yn-3-ol (No. 7) and (3R)-14-methyldocos-(4E)-en-1-yn-3-ol (No. 8) (Figure 2), which were isolated from the marine sponge *Cribrochalina vasculum* as the IGF-1R inhibitors in a tumor type selective manner. Silico docking and cellular thermal shift assay (CETSA) confirmed that compound **7** was bound to the kinase domain of IGF-1Rβ in NSCLC cells. Both compounds **7** and **8** impaired IGF-1Rβ phosphorylation and caused IGF-1Rβ degradation, and thereby led to activation of the intrinsic apoptotic pathway [24].

#### 2.1.3. Cyclin-Dependent Kinases (CDKs)

The cyclin-dependent kinases (CDKs) belong to a family of serine-threonine protein kinases whose activities are required for the cell cycle, and which are misregulated in 60–70% of human cancers [25]. Hymenialdisine and debromohymenialdisine (No. 9) (Figure 2), isolated from the marine sponge *Stylotella aurantium*, could inhibit cyclin-dependent kinases through competitive inhibition at the ATP-binding site. These two compounds were known to be active in a wide range of CDKs, particularly CDK1, CDK2, and CDK5. Hence, they were characterized by poor selectivity [26,27]. Another marine natural product, fascaplysin (No. 10) (Figure 2), isolated from the marine sponge, was a selective inhibitor of CDK4 with IC_50_ value of 0.35 μM. Fascaplysin was proved to selectively inhibit CDK4 by performing kinase activity assay using purified CDK-cyclin complexes. Molecular modelling suggested that fascaplysin inhibited CDK4 by binding to the ATP pocket of the kinase. Fascaplysin could inhibit the proliferation of endothelial cells and prevent angiogenesis, which suggested that it could be a leading compound for development of anticancer drug in the future [25,28]. Meridianins A–G (No. 11) (Figure 2), a group of marine indole alkaloids consisting of an indole framework connected to an aminopyrimidine ring, were isolated from marine tunicate *Aplidium meridianum* and found to potently and selectively inhibit CDK1, CDK5, and other various protein kinases involved in cancer and Alzheimer’s disease [29]. Computer-aided drug discovery design (CADD) techniques showed that meridianins A–G were bound to the ATP binding site of protein kinases, and acted as ATP competitive inhibitors [29,30].

#### 2.1.4. Glycogen Synthase Kinase-3 Beta (GSK-3β)

**Figure 3:**
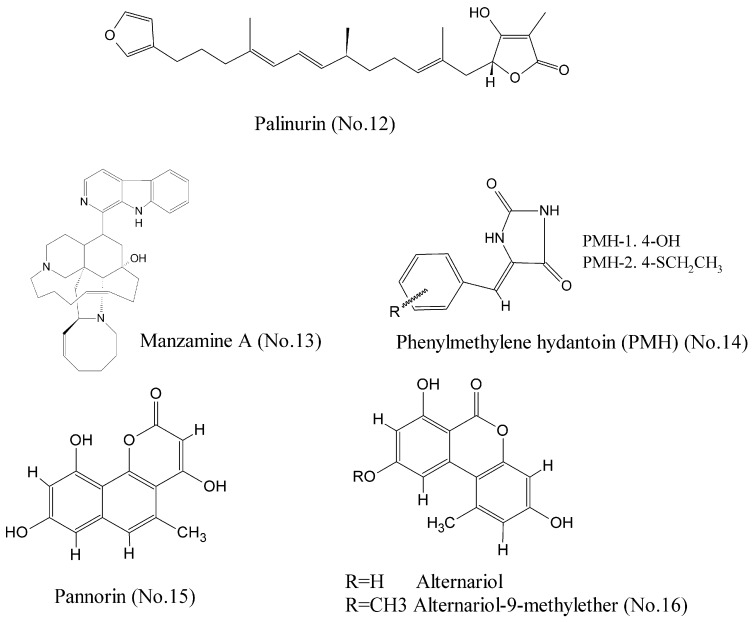
Compounds targeting GSK-3β.

Glycogen synthase kinase-3 beta (GSK-3β), a serine/threonine protein kinase that has been extensively implicated in critical cell biology processes, is a promising multipurpose kinase for cancer therapeutic target [31]. Bidon-Chanal et al. [32] characterized a marine natural sesquiterpene palinurin (No. 12) (Figure 3) as an ATP non-competitive GSK-3β inhibitor. Molecular modelling techniques proposed an unconventional binding mode through binding to the allosteric site of GSK-3β. It was the first compound to target this allosteric site, offering a new opportunity for designing and developing selective inhibitors with novel mechanisms of action. Manzamine A (No. 13) (Figure 3), a complex alkaloid isolated from a common Indonesian sponge *Acanthostrongylophora*, was shown to be a specific non-competitive inhibitor of ATP with binding to GSK-3β at IC_50_ value of 10.2 μM [33,34]. Studies of structure-activity relationship revealed that manzamine A was constituted of a promising scaffold for more potent and selective GSK-3β inhibitors. Additionally, molecular modeling study showed that phenylmethylene hydantoin (PMH-1) and the synthetic (Z)-5-(4-(ethylthio) benzylidene)-hydantoin (PMH-2) (No. 14) (Figure 3) from the Red Sea sponge *Hemimycale arabica* could be successfully docked into the binding pocket of GSK-3β. PMH reduced breast tumor growth and suppressed Ki-67, CD31, p-Brk, and p-FAK expression in tumor samples. Thus, it is a potential anticancer compound for the control of invasive breast malignancies [35]. Wiese et al. [36] reported that pannorin (No. 15), alternariol, and alternariol-9-methylether (No. 16) (Figure 3) were promising inhibitors of the isoform GSK-3β with nanomolar IC_50_ values, and had a highly oxygenated benzocoumarin core structure in common. Their study provided a new structural feature for efficient GSK-3β inhibition.

#### 2.1.5. Multi-Target Inhibitors of Receptor Tyrosine Kinases

**Figure 4:**
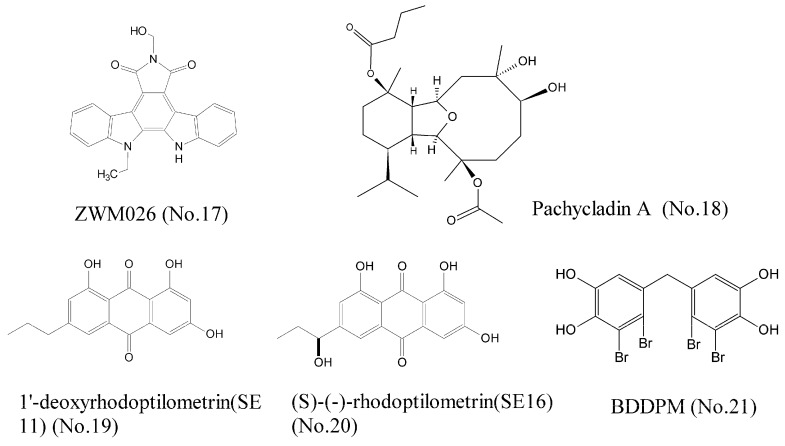
Compounds of multi-target receptor tyrosine kinases.

Cancer is a heterogeneous disease driven by many aberrant oncoproteins related to multiple pathways of signal transduction. Thus, development of multi-target agents is an urgent quest for the treatment of cancer. We recently found that ZWM026 (No. 17) (Figure 4), an indolocarbazole analogue derived from mangroves in coastal marine wetland, exhibited selectivity against T790M mutant (which is related to drug acquired resistance) over wild-type EGFR in NSCLC cells, and simultaneously inhibited activities of ErbB2, ErbB3, ErbB4, and RET, which were detected by kinase activity assay. Molecular docking experiment showed that the indolocarbazole rings of ZWM026 had hydrophobic interactions with the Leu718, Val726, Ala743, Met790, Glu791, Met793, and Leu844 of T790M mutant EGFR. ZWM026 more potently and selectively inhibited the growth of EGFR T790M mutant cells than wild-type EGFR cells, indicating that ZWM026 was a promising compound that could overcome drug acquired resistance [37]. Pachycladins, a group of diterpenoids, isolated from the Red Sea soft oral *Cladiella* species, significantly inhibited the drug-resistant T790M mutant EGFR and protein kinase C (PKC) [38]. However, pachycladin A (No. 18) (Figure 4) simultaneously inhibited the activity of wild-type EGFR. Molecular modeling assay elucidated that the oxabicycloundecane ring of pachycladin A could bind at the ATP pocket of EGFR kinase, either wild-type EGFR or mutant EGFR. Therefore, pachycladin A is not selective for wild-type EGFR and mutant EGFR, resulting in greater toxic side effects and a narrow therapeutic window, so it is necessary for the further structural modifications of this compound. Wätjen et al. [39] investigated antitumor effects of the anthraquinone derivatives 1′-deoxyrhodoptilometrin (SE11) (No. 19) and S-rhodoptilometrin (SE16) (No. 20) (Figure 4) in glioma and colon carcinoma cell lines, which were isolated from the marine echinoderm *Comanthus* sp. Results of kinase activity assay showed that these two compounds were potent inhibitors of IGF-1R, FAK, EGFR, ErbB2, and ErbB4. Wang et al. [40] reported that BDDPM (No. 21) (Figure 4), a bromophenol isolated from marine red alga *Rhodomelaceae confervoides*, was a potent multi-target receptor tyrosine kinase (RTK) inhibitor. Kinase activity assay revealed that BDDPM inhibited the activities of FGFR2, FGFR3, VEGFR2, and PDGFRα. It also down-regulated the phosphorylation of PKB/Akt and eNOS, as well as NO production. All these results indicated that BDDPM could be exploited as a novel multi-target RTK inhibitor [41]. 

### 2.2. Targeting Transcription Factors Related to Cancer Gene Expression

Transcription factor is a protein that binds to specific DNA sequence and regulates gene expression by promoting or suppressing transcription, which plays an important role in the occurrence, development, infiltration, and metastasis of tumor.

**Figure 5:**
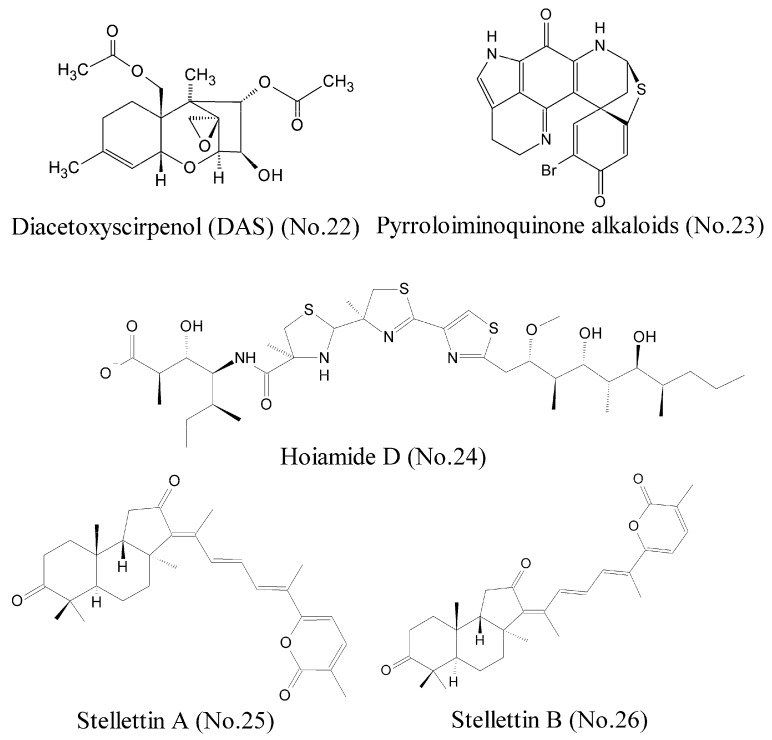
Compounds targeting transcription factor.

Hypoxia-inducible factor 1 (HIF-1), which is generally regarded as a tumor prospective factor related to tumor cell proliferation, apoptosis, metabolism, and angiogenesis, is one of the most compelling targets for treating cancers [42]. Choi et al. [43] identified a compound, diacetoxyscirpenol (DAS) (No. 22) (Figure 5), which originated from a marine bacterium living on red alga, contained the 12, 13-epoxytrichothecene group of sesquiterpenes as the core structure, and inhibited HIF-1 expression and its transcriptional activity in cancer cells exposed to hypoxia. Luciferase reporter assay showed that DAS inhibited de novo synthesis of HIF-1α protein by blocking the 5′-UTR-mediated translation of HIF-1α mRNA. Furthermore, DAS interfered with the dimerization of HIF-1α and ARNT (aryl hydrocarbon receptor nuclear translocator), which might be attributed to impair nuclear translocation of HIF-1α. Animal experiments demonstrated that DAS inhibited the growth of lung carcinoma xenografts in mice. Pyrroloiminoquinone alkaloids (No. 23) (Figure 5) from the marine sponge *Latrunculia* sp., which were identified as novel HIF-1α/p300 inhibitors, interrupted the protein-protein interaction between HIF-1α and p300 [44], and potently inhibited the growth of HCT 116 and prostatic carcinoma cell lines in vitro models.

A lot of evidence shows that MDM2 is an oncogene, and it can bind to p53 and inhibit the functions of p53 [45]. Thus, disruption of any of these regulatory functions by MDM2 is a viable strategy to reactivate p53, especially through inhibition of the p53/MDM2 binding interaction. Hoiamide D (No. 24) (Figure 5), a marine cyanobacteria-derived polyketide compound that featured two consecutive thiazolines (thiazoles and isoleucine residues), displayed inhibitory activity against p53/MDM2 interaction [46]. The inducible transcription factor, NF-κB, plays an important role in the regulation of immune, inflammatory, and carcinogenic responses, and has become a major molecular target in drug discovery. NF-κB is a dimer of proteins belonging to the Rel family, which includes RelA (p65), RelB, c-Rel, p50 (NF-κB1), and p52. One strategy is to interfere with the binding of NF-κB to DNA. Such a compound as gallic acid, for example, can inhibit NF-κB activation by impeding the binding of p50 to DNA specifically [47]. Folmer et al. [48] purified and characterized many compounds from different marine sponges and soft corals, and found that stellettin A (No. 25) and stellettin B (No. 26) (Figure 5) had potent inhibition to NF-κB by inhibiting the binding of p50/p65 to DNA. These two compounds possessed lactone rings with α, β-unsaturated carbonyl groups that played a major role in the inhibition activity. Both compounds inhibited activation of NF-κB by inducing an overexpression of IKKβ, which resulted in a cytotoxic effect on the human leukemia cell line K562.

### 2.3. Targeting Histone Deacetylases Related to Epigenetic Regulation of Cancer

**Figure 6:**
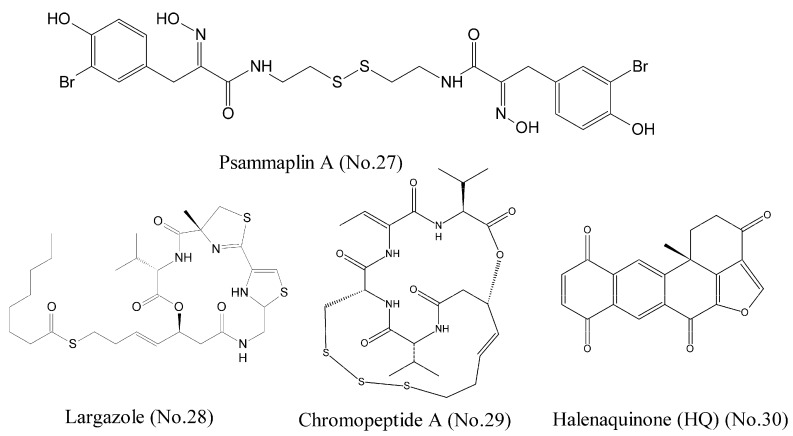
Compounds targeting HDACs.

Histone deacetylases (HDACs) are a class of enzymes that remove acetyl groups from an N-acetyl lysine amino acid on a histone and allow the histones to wrap the DNA more tightly. The dysregulation of DNA methylation and acetylation of the lysine residues on histone tails generally result in genomic instability of tumor [49]. Psammaplin A (No. 27) (Figure 6), which was isolated from several marine sponges including *Pseudoceratina purpurea*, was reported to be a potent inhibitor of HDAC [50,51]. The results of fluorogenic histone deacetylase assay demonstrated that psammaplin A had high isoform selectivity and 360-folds selective for HDAC1 (IC_50_ is 0.9 nM) over HDAC6. Psammaplin A could release a free thiol function as a zinc binding group, and the studies of structure-activity relationship suggested the requirement of a free oxime for potent HDAC1 inhibition. The computational docking studies and molecular dynamics simulations illustrated that psammaplin A could form three hydrogen bridges to Y303, D99, and the protonated H141. Largazole (No. 28) (Figure 6) was originally discovered from the Floridian marine cyanobacterium *Symploca* sp. and produced a novel cyclic depsipeptide that was a potent HDAC inhibitor. Largazole was a prodrug and generated largazole thiol that could interact with the zinc ion in the active site of HDACs. Molecular docking studies showed that largazole thiol, as well as analogs of largazole thiol, docked into a homology model of HDAC1. Largazole thiol was more active against recombinant HDAC1 than any other marine-derived HDAC inhibitor; for example, psammaplin A. Largazole inhibited HDACs in tumor tissue of a human HCT116 xenograft mouse [52]. 

Chromopeptide A (No. 29) (Figure 6), a depsipeptide isolated from the marine sediment-derived bacterium, was identified as a novel HDAC inhibitor. HDAC enzyme selectivity and kinetic analysis showed that chromopeptide A selectively inhibited HDAC1, 2, 3, and 8 in a non-competitive manner. Cellular experiments demonstrated that it dose-dependently suppressed the proliferation and the migration of human prostate cancer cell lines PC3, caused cell cycle arrest, and induced cell apoptosis. Moreover, chromopeptide A significantly suppressed the tumor growth in mice bearing PC3 prostate cancer xenografts [53]. Halenaquinone (HQ) (No. 30) (Figure 6), a marine polycyclic quinone-type metabolite, acted as an HDAC and topoisomerase inhibitor. HQ inhibited deacetylation of HDAC activity through a cell-free HDAC colorimetric acetylated lysine side chain assay using an enzyme-mediated deacetylation. The results of western blotting indicated that HQ could inhibit the expression of anti-apoptotic proteins p-Akt, NF-κB, and Bcl-2 [54,55]. As the structure of halenaquinone (HQ) does not contain sulfur moiety, it is speculated that the mechanism of HDAC inhibition was different from those of the three compounds mentioned above.

### 2.4. Targeting Proteasome and Deubiquitylating Enzymes Related to Oncoprotein Degradation

In the ubiquitin-proteasome system, a majority of cellular proteins are degraded by the proteasome pathway related to three enzymes: ubiquitin-activating enzyme (E1), ubiquitin-conjugating enzyme (E2), and ubiquitin-protein ligase (E3) [56]. Given that many proteins in the ubiquitin-proteasome system are involved in the regulation of important processes of carcinogenesis, targeting the ubiquitin-proteasome system has been a therapeutic strategy in clinical treatment of cancer. 

**Figure 7:**
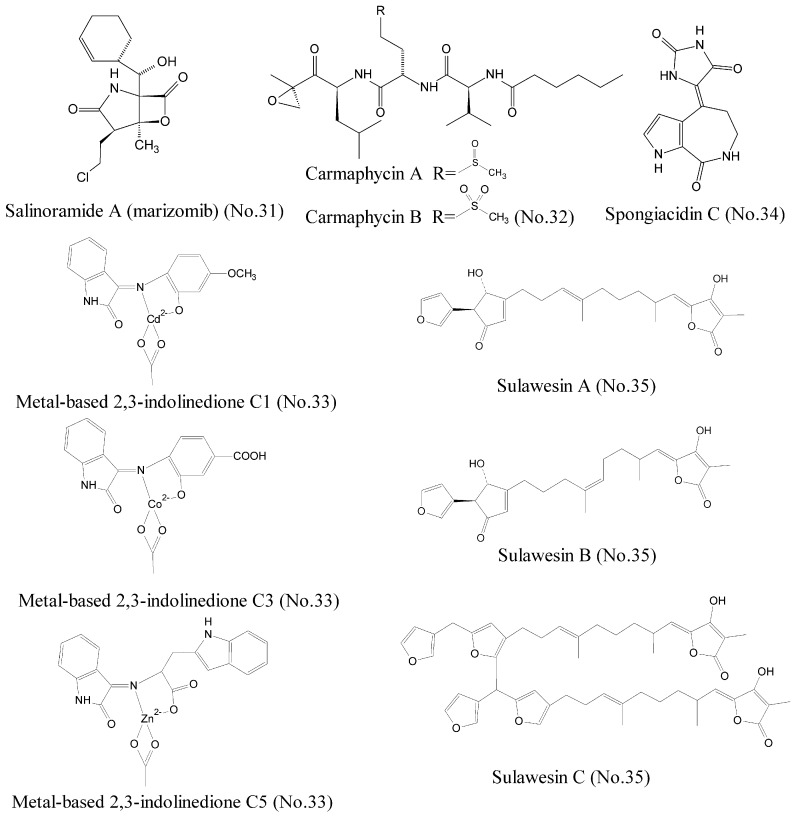
Compounds targeting proteasome and deubiquitylating enzymes.

To date, a great deal of effort has been devoted to searching proteasome inhibitors for the treatment of cancer. It has been established that the first generation of proteasome inhibitor bortezomib is effective as monotherapy treatment of hematologic malignancies such as multiple myeloma [57,58]. Salinosporamide A (marizomib) (No. 31) (Figure 1), isolated from a new marine actinomycete bacteria *Salinispora tropica* in ocean sediments, was widely reported as a novel 20S proteasome inhibitor for treatment of cancer [59,60]. Salinosporamide A possessed a densely functionalized γ-lactam-β-lactone bicyclic core, which was responsible for its irreversible binding to its target, the β∣ subunit of the 20S proteasome. Salinosporamide∣ A has entered into phase I clinical trials as monotherapy for the treatment of multiple myeloma, as well as other solid tumor and hematologic malignancies [61,62]. Further studies suggested that salinosporamide A in combination with chemotherapeutics, such as vorinostat, enhanced the curative efficiency against some refractory melanoma, pancreatic carcinoma, and NSCLC. Recently, it was reported that carmaphycin A and carmaphycin B (No. 32) (Figure 7), which were isolated from a Curaçao collection of marine cyanobacteria *Symploca* sp., had potent anti-proteasome properties as potential therapeutic agents for treatment of cancer [63]. Carmaphycins feature a leucine-derived α, β-epoxyketone warhead that is directly connected to either methionine sulfoxide or methionine sulfone. Simulations of molecular dynamics demonstrated that the sulfoxide/sulfone moieties in the methionine-derived residues could bind to the NH group of Gly23 with the hydrogen bond, proposing a new distinctive binding mode for these inhibitors. In addition, metal-based 2, 3-indolinedione derivatives (No. 33) (Figure 7), which existed in marine organisms, were reported to inhibit proteasome activity and induce apoptosis in certain human cancer cells. These novel metal-based complexes with derivatives of 2,3-indolinedione inhibited the chymotrypsin-like activity of the human cancer cellular 26S proteasome and promoted the accumulation of the proteasome target protein Bax due to their unique structures [64]. The studies of structure-activity relationship revealed that the aromatic ring with electron-attracting capabilities could transport metal into cancer cells more easily by changing the electron density and nucleophilic attack.

The ubiquitylation of protein is reversed by deubiquitylating enzymes (DUBs), and leads to deconjugation of the ubiquitin chain [65]. To date, nearly 100 species of human DUBs have been found, including ubiquitin specific peptidase 7 (USP7), which affects the stability and degradation of cellular proteins [66]. USP7 is an emerging oncology target, because it involves the oncogenic stabilization of the tumor suppressor protein, p53 [67]. USP7 can deubiquitylate Hdm2 and consequently degrade p53. Hence, inhibiting USP7 stabilizes p53 in cells through degradation of Hdm2 and subsequently results in the suppression of cancer [68]. Spongiacidin C (No. 34) (Figure 7), a pyrrole alkaloid, was isolated from the marine sponge *Stylissa massa* and identified as the first USP7 inhibitor [69]. Compared to some previously described USP7 inhibitors derived from synthetic sources, spongiacidin C exhibited a higher potent inhibition activity of USP7 with an IC_50_ of 3.8 μM. In addition, three new furanosesterterpene tetronic acids, sulawesins A–C (No. 35) (Figure 7) from marine sponge *Psammocinia* sp., which possessed a new carbon skeleton with a 5-(furan-3-yl)-4-hydroxycyclopent-2-enone moiety, were found to inhibit USP7 with IC_50_ values in the range of 2.7–4.6 μM [70].

### 2.5. Targeting the Heat Shock Protein (Hsp90) Related to Cancer Oncoprotein Maturity

**Figure 8:**
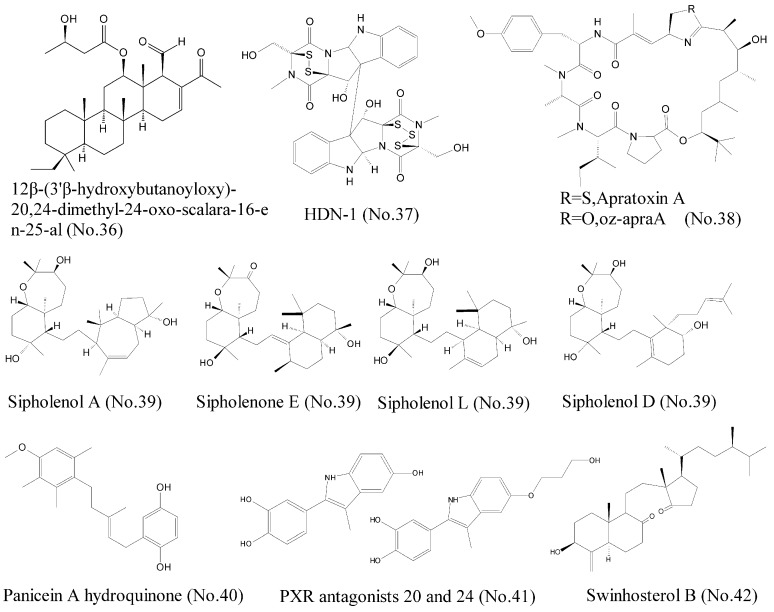
Compounds targeting Hsp90, P-gp, Patched, and PXR.

Heat shock protein 90 (Hsp90) functions as an evolutionarily conserved molecular chaperone and plays an essential role in cell survival, proliferation, apoptosis, and cellular homeostasis [71]. A growing body of evidence indicates that Hsp90 is frequently unregulated in many solid tumors, including lung cancer, breast cancer, colorectal cancer, and hematological malignancy. Consequently, Hsp90 has been recognized as a crucial target in cancer treatment, and increasing number of small molecule inhibitors of Hsp90 have been identified. Lai et al. [72] reported that three terpenoids, 12β-(3′β-hydroxybutanoyloxy)-20, 24-dimethyl-24-oxo-scalara-16-en-25-al (No. 36) (Figure 8), which were isolated from the sponge *Carteriospongia* sp., induced apoptosis via dual inhibitory effects on Hsp90 and topoisomerase II against leukemia cells. Molecular docking analysis showed that the compound was bound to N-terminal ATP-binding pocket of Hsp90 protein and promoted degradation of Hsp90 client proteins such as Akt, Raf-1, CDK4, Cyclin D3, HIF 1, and HSF1. HDN-1 (No. 37) (Figure 8), an epipolythiopiperazine-2, 5-diones (ETPs) compound, was isolated from the Antarctic fungus *Oidiodendron truncatum* GW3-13 and identified as a new Hsp90 inhibitor. Surface plasmon resonance and molecular docking experiments revealed that HDN-1 was bound directly to C-terminus of Hsp90α, and led to the potent inhibition of cell survival and proliferation by downregulating various protein expressions [71]. Additionally, an oxazoline analogue of apratoxin A (oz-apraA) (No. 38) (Figure 8), structurally characterized by cyclodepsipeptide, was isolated from a marine cyanobacterium and promoted the degradation of Hsp90 clients through chaperone-mediated autophagy [73]. Apratoxin A inhibited Hsp90 function by stabilizing the interaction of Hsp90 client proteins with Hsc70/Hsp70 and thus prevented their interactions with Hsp90. 

### 2.6. Targeting P-gp, Patched, and PXR Related to the Cancer Multidrug Resistance

P-glycoprotein (P-gp) is known as multidrug resistance 1 (MDR1) or ATP-binding cassette sub-family B member 1 (ABCB1), and belongs to ABC transporter family. This family also includes ABCG2/breast cancer resistance protein (BCRP), which is associated with multidrug resistance (MDR) [74]. Therefore, exploitation of anticancer leading compounds, which could inhibit these ABC transporter proteins, is an effective approach to reverse resistance and further improve therapeutic efficacy. Abraham et al. [75] summarized several marine natural products with reversal effects on multidrug resistance in cancer. Sipholane triterpenoids (No. 39) (Figure 8), which were derived from the Red Sea sponge *Callyspongia siphonella*, represented potential reversal agents for the treatment of MDR in P-gp-overexpressed tumors [76]. These sipholane triterpenoids efficiently inhibited the function of P-gp through direct interaction rather than alteration of the expression of P-gp. Aller et al. [77] identified three binding sites in the crystallographic structure of P-gp, which were QZ59-RRR, QZ59-SSS, and verapamil binding sites. Molecular docking techniques showed that these compounds were docked at each binding sites. Sipholenone E showed a hydrogen bonding interaction of C-10 hydroxyl group with the Gln 721 which may explain its higher binding score [76]. Other marine natural products, such as agosterol A, ET-743, bryostatin 1, welwitindolinones, philinopside A, and philinopside E, also inhibited drug efflux through targeting P-gp and MRP1 and thus reversed the resistance [75,76]. 

Several studies have shown that Hh receptor Patched has activity to mediate drug efflux and participates in chemotherapy resistance, indicating that it is a new target for anti-cancer therapy [78]. Consequently, compounds that inhibit the drug efflux against Patched can increase the efficiency of chemotherapy and reduce the possibilities of recurrence of cancer. Based on discovery of a class of natural compounds from Mediterranean sponge *Haliclona (Soestella) mucosa*, Fiorini et al. [79] found that panicein A hydroquinone (No. 40) (Figure 8) inhibited the multidrug resistance activity of Patched and increased chemotherapy efficiency on melanoma cells. Molecular docking model showed that panicein A hydroquinone presented a strong docking cluster close to the doxorubicin binding site of Patched, suggesting that panicein A hydroquinone and doxorubicin competed the similar binding sites in Patched. Therefore, the compound appeared to be the first antagonist of Patched to block drug efflux. Patched efflux inhibitors can be used by combining with classic chemotherapy to represent a new way to reduce tumor resistance, relapse, and metastasis [79].

The pregnane X receptor (PXR) regulates the expression of efflux ATP-binding cassette (ABC) drug transporters such as P-gp, MRP1, and BCRP, indicating the importance of PXR as a drug target for countering multidrug resistance in cancer treatments. ET-743 (No. 2) (Figure 1), previously mentioned as a potent antineoplastic agent, was reported as the first PXR antagonist that could suppress paclitaxel-induced PXR activation [80]. Later, Hodnik et al. [81] discovered that bazedoxifene scaffold-based compounds, inspired by the marine sulphated steroids solomonsterols A and B, were novel PXR antagonists. PXR antagonists 20 and 24 (No. 41) (Figure 8) were found to inhibit PXR-mediated drug metabolism by inhibiting PXR expression. Molecular docking experiments showed that these compounds could interact with the ligand-binding site of PXR. Interestingly, swinhosterol B (No. 42) (Figure 8) from *Theonella swinhoei* sponge was reported as a natural PXR agonist and an FXR antagonist. The molecular docking results showed that this compound also interacted with PXR ligand binding pocket by hydrogen and van der Waals bonds [82].

### 2.7. Compounds Targeting Other Cancer Related Molecules

Except for those target molecules mentioned above, we also reviewed other novel molecular targets of marine-derived compounds that were studied in preclinical trial, including ion channel, RNA helicase eIF4A, ribosome, TRPM 7, and so on.

**Figure 9:**
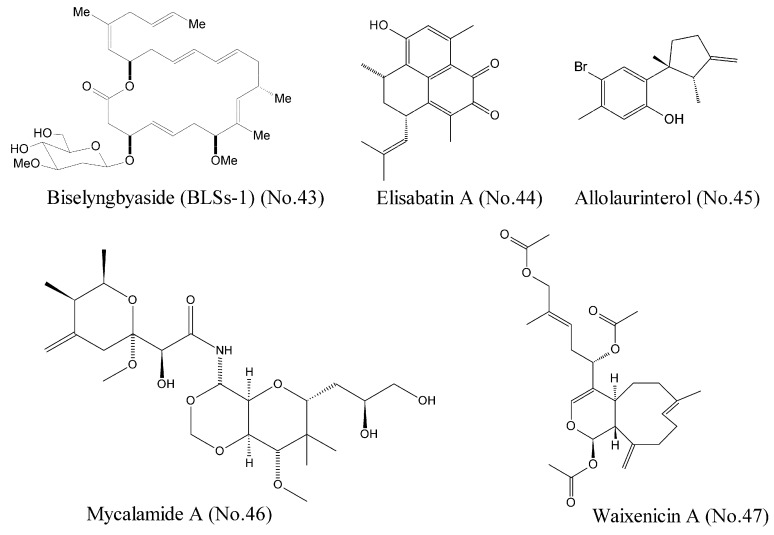
Compounds targeting other cancer related molecules.

Morita et al. [83] reported that Biselyngbyaside (BLSs-1) (No. 43) (Figure 9), a macrolide from a marine cyanobacterium, was a high affinity (the affinity constant Ki was 10 nM) inhibitor of Ca^2+^ pumps with a unique binding mode. The crystal structures and activity measurement of BLSs-1 showed that BLSs-1 was bound to the pump near the cytoplasmic surface of the transmembrane region and displayed potent cytotoxicity against a variety of human cancer cells. 

DEAD box RNA helicase eIF4A is an ATP-dependent helicase involved in RNA metabolism. It is a potential therapeutic target for a variety of malignancies [84]. Tillotson et al. [84] reported that marine-derived natural products such as elisabatin A (No. 44) and allolaurinterol (No. 45) (Figure 9) potently inhibited eIF4A in an ATP competitive manner, which was detected by enzymological analyses. These two compounds were most likely bound to the ATP-binding pocket at the interface between the N-terminal and C-terminal domains. Cellular evaluations showed their potent cytotoxicity against A549 and MDA-MA-468 cell lines. Both compounds potently inhibited eIF4A ATPase activity, but only allolaurinterol showed potent inhibition of helicase activity.

Mycalamide A (No. 46) (Figure 9), a marine natural compound isolated from sponges of the genus *Mycale*, was known as a protein synthesis inhibitor with potent antitumor activity. This compound inhibited transcriptional activity of the oncogenic nuclear factors AP-1 and NF-κB and induced the phosphorylation of the kinases MAPK p38, JNK, and ERK, indicating a promising potential for both cancer-prevention and cytotoxic therapy [85]. Binding experiments demonstrated that mycalamide A could bind to the large ribosomal subunit and inhibit translation of RNA into protein [86].

Zierler et al. [87] identified waixenicin A (No. 47) (Figure 9) from the soft coral *Sarcothelia edmondsoni* as the first potent and relatively specific inhibitor of TRPM7 ion channels. Potential transient receptor melastatin 7 (TRPM7) channel, a bifunctional membrane protein with ion channel and kinase activity, represents the major magnesium-uptake mechanism in mammalian cells and is a key regulator of cell growth and proliferation [88]. Mutational analysis involving the channel kinase domain revealed that waixenicin A could be bound to TRPM7 outside of the kinase domain with high affinity and independently blocked the channel of Mg^2+^, which was responsible for the relatively specificity of TRPM7 [87]. 

Marine-derived compounds that can modulate the activity of molecular targets involved in tumorigenesis, and their molecular targets enrolled in this article, are shown in Table 1.

## 3. Conclusions

Although the past decades have witnessed intensive efforts to exploit leading compounds with anticancer activities from marine microorganisms, many molecular targets of these candidates remain elusive. Illumination of molecular targets of leading compounds will contribute to mechanism clarification, as well as improvement of drug ability. 

In recent years, more new technologies such as biochips technology, chemical proteomics approaches, and CRISPR/Cas9 high-throughput screening technology have been used to identify targets of a number of new compounds. In addition, drug-target prediction with silico technology can quickly predict potential molecular targets based on a database containing a large number of potential targets and bioactive compounds with definite molecule structures using molecular docking. The computer-aided drug discovery design (CADD) technique has been applied to provide precise information regarding the binding mode against molecular targets, which may contribute to the development of antitumor drugs in the future.

This paper reviews marine-derived compounds that can modulate the activity of molecular targets involved in tumorigenesis. We hope the review could provide help for target identification of new compounds in the future. Of course, on account of their novel structures and unconventional anticancer molecular mechanisms, these marine candidates are undoubtedly attractive as leading compounds. Therefore, the development of anticancer drugs needs further investigation.

We believe that an increasing number of molecular targets will be clarified in the near future with the advance in drug screening and identification techniques. Thus, it is certain that the future chemotherapeutic clinical pipeline will be fed with marine-derived agents, which paves the way for curing cancer and benefiting human health.

## Figures and Tables

**Table 1:** List of marine-derived compounds that have exhibited potential as cancer therapies.

No.	Compound Name	Marine Organism	Chemical Class	Molecular Target	Cancer Type/Cell lines	Refs.
5	Bryostatin-1	bryozoan	oxygenated macrolide	PKC activator	sarcoma, melanoma, ovaria, cervical, neck and head carcinoma, esophageal, gastric, pancreatic, renal cell carcinoma, leukemia cells	[16,17,18]
6	Aplysiatoxin (ATX)	sea hare and cyanobacteria	polyacetate	PKC activator	Leukemia cell, breast cancer cell	[19]
7,8	(3R)-icos-(4E)-en-1-yn-3-ol and (3R)-14-methyldocos-(4E)-en-1-yn-3-ol	marine sponge	acetylene alcohols	IGF-1Rβ	NSCLC cells	[23,24]
9	Hymenialdisine and Debromohymenialdisine	marine sponge	pyrrole-2-aminoimidazole alkaloids	CDK1, CDK2, CDK5,	colon carcinoma cell lines LoVo and Caco-2	[26,27]
10	Fascaplysin	marine sponge	carboline class alkaloid	CDK4	osteosarcoma U2OS, colon carcinoma cell HCT116	[25,28]
11	Meridianin A-G	marine tunicate	indole alkaloids	CDK1, CDK5	/	[29,30]
12	Palinurin	marine sponge	linear furanosesquiterpene	GSK-3β	human neuroblastoma cells SH-SY5Y	[32]
13	Manzamine A	marine sponge	alkaloid	GSK-3β	pancreatic cancer cell	[33,34]
14	PMH-1 and PMH-2	marine sponge	cyclic imide hydantoins	GSK-3β	prostate cancer cell	[35]
15	Pannorin	marine fungi	oxygenated benzocoumarin core	GSK-3β	/	[36]
16	Alternariol, and Alternariol-9-methylether	marine fungi	oxygenated benzocoumarin core	GSK-3β	/	[36]
17	ZWM026	mangrove	indolocarbazoles	EGFR-T790M, ErbB2, ErbB3, ErbB4, and RET	lung cancer cells	[37]
18	Pachycladin A	Red Sea soft coral	diterpenoids	EGFR and PKC	breast cancer cell lines, cervical cancer HeLa cells	[38]
19,20	1’-deoxyrhodoptilometrin (SE11) and (S)-(−)-rhodoptilometrin (SE16)	marine echinoderm	anthraquinone	IGF-1R, FAK, EGFR, ErbB2, and ErbB4	glioma and colon carcinoma	[39]
21	BDDPM	marine red alga	bromophenol	FGFR2,3,VEGFR2,PDGFRα,PKB/Akt,eNOS	hepatoma carcinoma cell	[40,41]
22	Diacetoxyscirpenol (DAS)	marine red alga bacterium	enol	HIF-1α	lung cancer cell lines A549	[43]
23	Pyrroloiminoquinone alkaloids	marine sponge	alkaloids	HIF-1α/p300	colon and prostatic carcinoma	[44]
24	Hoiamide D	marine cyanobacteria	polyketide	p53/MDM2	lung cell lines H460	[46]
25,26	Stellettin A and Stellettin B	marine sponge	triterpenoids	p50/p65	Leukemia cell line K562	[48]
27	Psammaplin A	marine sponge	indole	HDAC1	lung, breast cancer cell lines	[50,51]
28	Largazole	marine cyanobacterium	cyclic depsipeptide	HDAC1	colon cancer cell lines HCT116	[52]
29	Chromopeptide A	marine bacterium	depsipeptide	HDAC1,2,3,8	prostate cancer cell lines PC3	[53]
30	Halenaquinone (HQ)	marine sponge	polycyclic quinone-type	HDACs	Molt 4, K562, MDA-MB-231, and DLD-1 cell lines	[54,55]
31	Salinosporamide A	marine actinomycete bacteria	γ-lactam-β-lactone bicyclic core	20S proteasome	melanoma, pancreatic carcinoma, or NSCLC	[59,60,61,62]
32	Carmaphycin A and carmaphycin B	marine cyanobacteria	leucine-derived α,β-epoxyketone	proteasome	lung and colon cancer cell lines	[63]
33	Metal-based 2, 3-indolinedione	marine organisms	metal-based complexes with derivatives of 2,3-indolinedione	26S proteasome	breast cancer cell lines MDA-MB-231 and prostate cancer cell lines LNCaP and PC-3	[64]
34	Spongiacidin C	marine sponge	pyrrole alkaloid	USP7	/	[69]
35	Sulawesins A–C	marine sponge	furanosesterterpene tetronic acids	USP7	/	[70]
36	12β-(3′β-hydroxybutanoyloxy)-20, 24-dimethyl-24-oxo-scalara-16-en-25-al	marine sponge	sesterterpenoids	Hsp90	Leukemia cell lines	[72]
37	HDN-1	antarctic fungus	epipolythiopiperazine-2, 5-diones (ETPs)	Hsp90	lung cancer cell lines	[71]
38	Apratoxin A (oz-apraA)	marine cyanobacterium	cyclodepsipeptide	Hsp90	A549, MDA-MB-453, HEK293, SKoV3, and H4 cells	[73]
39	Sipholane triterpenoids	marine sponge	perhydrobenzoxepine ring and a bicyclodecane system	P-gp	human oral epidermoid carcinoma cell line KB-C2 and KB-V1	[75,76]
40	Panicein A hydroquinone	marine sponge	hydroquinone	Patched	melanoma cells	[79]
41	PXR antagonists 20 and 24	sponges and echinoderms	Sulfated steroids	PXR agonist	HepG2 cells	[81]
42	Swinhosterol B	marine sponge	4-methylenesterols	PXR agonist	HepG2 cell	[82]
43	Biselyngbyaside (BLSs-1)	marine cyanobacterium	macrolides	calcium channel	HeLa cells	[83]
44	Elisabatin A	Indian gorgonian octocoral	polyketone	eIF4A ATPase activity	A549 and MDA-MA-468 cell lines	[84]
45	Allolaurinterol	marine red alga	benzene derivative	eIF4A ATPase activity	A549 and MDA-MA-468 cell lines	[84]
46	Mycalamide A	marine sponge	lactones	protein synthesis inhibitor	JB6 Cl 41 P+, HeLa cell line	[85,86]
47	Waixenicin A	soft coral	polyketone	TRPM7	Jurkat and RBL cells	[87]
